# Is adipose tissue suitable for detection of (synthetic) cannabinoids? A comparative study analyzing antemortem and postmortem specimens following pulmonary administration of JWH-210, RCS-4, as well as ∆9-tetrahydrocannabinol to pigs

**DOI:** 10.1007/s00204-020-02843-x

**Published:** 2020-07-14

**Authors:** Nadine Schaefer, Frederike Nordmeier, Ann-Katrin Kröll, Christina Körbel, Matthias W. Laschke, Michael D. Menger, Hans H. Maurer, Markus R. Meyer, Peter H. Schmidt

**Affiliations:** 1 Institute of Legal Medicine, Saarland University, Building 49.1, 66421 Homburg, Germany; 2 Institute for Clinical and Experimental Surgery, Saarland University, Building 65/66, 66421 Homburg, Germany; 3 Department of Experimental and Clinical Toxicology, Center for Molecular Signaling (PZMS), Saarland University, Building 46, 66421 Homburg, Germany

**Keywords:** Synthetic cannabinoids, Tetrahydrocannabinol, Adipose tissue, postmortem redistribution, Pigs, Pulmonary administration

## Abstract

Examining fatal poisonings, chronic exposure may be reflected by the concentration in tissues known for long-term storage of drugs. Δ9-tetrahydrocannabinol (THC) persists in adipose tissue (AT), but sparse data on synthetic cannabinoids (SC) are available. Thus, a controlled pig study evaluating antemortem (AM) disposition and postmortem (PM) concentration changes of the SC 4-ethylnaphthalene-1-yl-(1-pentylindole-3-yl)methanone (JWH-210) and 2-(4-methoxyphenyl)-1-(1-pentyl-indole-3-yl)methanone (RCS-4) as well as THC in AT was performed. The drugs were administered pulmonarily (200 µg/kg body weight) to twelve pigs. Subcutaneous (s.c.) AT specimens were collected after 15 and 30 min and then hourly up to 8 h. At the end, pigs were sacrificed and s.c., perirenal, and dorsal AT specimens were collected. The carcasses were stored at room temperature (RT; *n* = 6) or 4 °C (*n* = 6) and specimens were collected after 24, 48, and 72 h. After homogenization in acetonitrile and standard addition, LC–MS/MS was performed. Maximum concentrations were reached 0.5–2 h after administration amounting to 21 ± 13 ng/g (JWH-210), 24 ± 13 ng/g (RCS-4), and 22 ± 20 ng/g (THC) and stayed at a plateau level. Regarding the metabolites, very low concentrations of *N*-hydroxypentyl-RCS-4 (HO-RCS-4) were detected from 0.5 to 8 h. PM concentrations of parent compounds did not change significantly (*p* > 0.05) over time under both storage conditions. Concentrations of HO-RCS-4 significantly (*p* < 0.05) increased in perirenal AT during storage at RT. These results suggest a rapid distribution and persistence in s.c. AT. Furthermore, AT might be resistant to PM redistribution of parent compounds. However, significant PM increases of metabolite concentrations might be considered in perirenal AT.

## Introduction

Interpreting postmortem (PM) drug concentrations in the context of death investigation, consumption habits, that is to say acute versus chronic consumption, have to be considered. Assessing analytical findings in tissues known for long-term persistence of substances concerning that issue might be helpful. Although a precise determination of the time of consumption is not possible, adipose tissue (AT) proved to be an alternative specimen for detecting drugs, pharmaceuticals, and pollutants, especially lipophilic ones, in PM toxicology (Colucci et al. 2010; De Saeger et al. 2005; Hikiji et al. 2010; Mühlebach et al. 1985; Shintani-Ishida et al. 2018). Contrary to blood, AT is available in higher amounts, even if a decedent is already putrefied.

Furthermore, a study by Levisky et al. (2001) provided data on drugs detected in PM AT specimens. Those results implicated that determination of drugs in this tissue prove an antemortem (AM) disposition not being affected by PM redistribution (PMR). However, this study was based on data from authentic autopsy cases and was burdened with the uncertainty that time of intake, dose and PM interval (PMI) were unknown.

∆^9^-Tetrahydrocannabinol (THC), a highly lipophilic drug, has already extensively been studied in terms of its toxicokinetic (TK) properties. It is well-known that THC is stored in AT. Kreuz and Axelrod (1973) demonstrated a persistence for 2 weeks after single dose administration and Johansson et al. (1989) detected THC in human AT specimens from heavy marihuana users 4 weeks after having smoked their last four cigarettes before sampling. Rawitch et al. (1979) examined the time-dependent uptake of THC into mouse AT of different regions following a single intraperitoneal injection. The authors found a preferential accumulation in gonadal fat. Brunet et al. (2010) investigated the PMR of THC in pigs following a single intravenous administration, but they did not determine the AM uptake of THC into AT.

The highly lipophilic synthetic cannabinoids (SC) should show a comparable distribution and persistence in the body, but so far very little is known about their TK and toxicodynamics (TD). A number of case reports have already been published discussing a contribution of SC to the fatal outcome and describing the (adipose) tissue distribution (Kraemer et al. 2019). However, those data should be interpreted with caution, because in most of the cases, dose and time of drug intake as well as time of death and PMI were unknown. Controlled systematic human studies are not allowed for ethical reasons, but TK studies are necessary for a better understanding of the toxicology of those substances. Thus, animal studies have to be performed.

Poklis et al. (2012b) studied the disposition of JWH-018 and JWH-073 in mouse blood and brain after exposure to “Magic Gold” smoke and also determined JWH-018 in different organs of the mouse after inhalation exposure to “buzz” smoke (Poklis et al. 2012a; Wiebelhaus et al. 2012). However, they did not assess SC concentrations in AT. In fact, data of only two studies on the distribution of SC AT in rodents are available. In the first study including three mice, 30% of the total intraperitoneally injected dose of the SC WIN55,212-2 were detected after 30 min in AT (Barna et al. 2009). In the second study we showed a storage of the SC JWH-122 and JWH-210 for at least four weeks following single oral administration to rats (Schaefer et al. 2014). However, these studies only included a small number of animals. In addition, no time-course of AM uptake into AT was investigated.

**Fig. 1:**
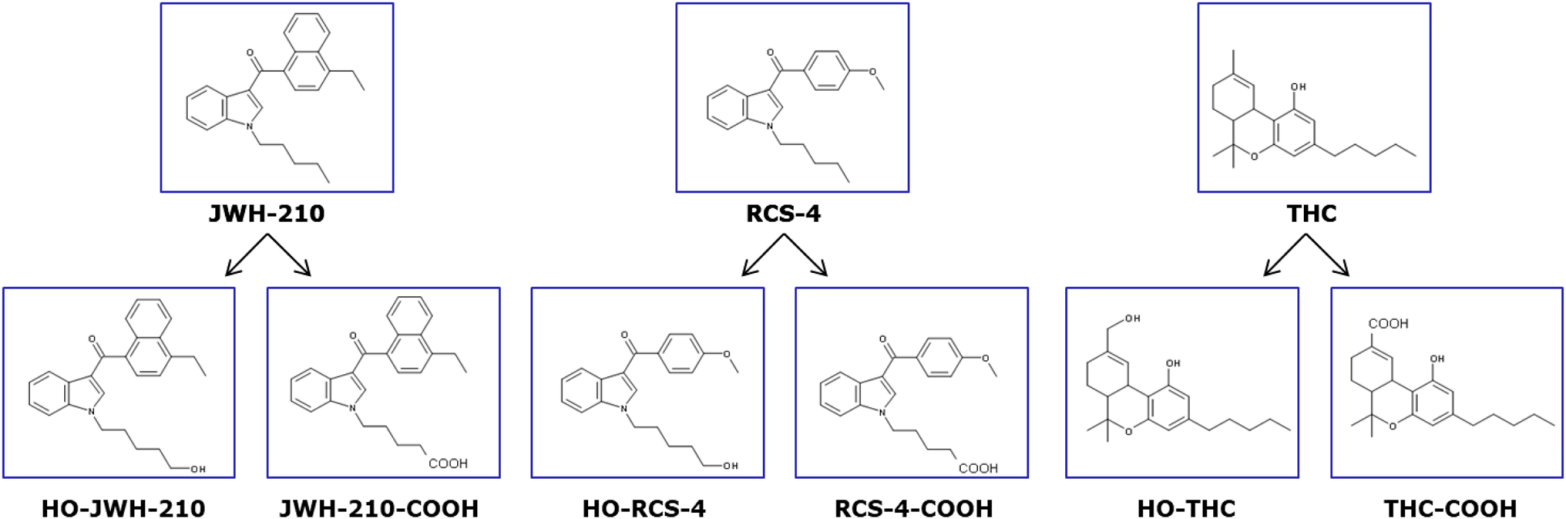
Molecular structures of the tested drugs and their main metabolites

Recently, we established a pig model allowing for elucidation of cannabinoid TK after pulmonary administration of 4-ethylnaphthalene-1-yl-(1-pentylindole-3-yl)methanone (JWH-210), 2-(4-methoxyphenyl)-1-(1-pentyl-indole-3-yl)methanone (RCS-4), and THC (Schaefer et al. 2018a, b). The chemical structures are depicted in Fig. 1. As part of the study, AM data on the distribution of the cannabinoids and their main metabolites (see Fig. 1) in blood (Schaefer et al. 2018a, b), perimortem data on the distribution in different organs at the time of death (Schaefer et al. 2019) as well as time- and temperature-dependent concentration changes were provided. Pigs were chosen, because they have already been proven to be suitable for the examination of PM concentrations of central nervous acting substances (Brunet et al. 2010; Crandall et al. 2006; Flanagan et al. 2003; Hilberg et al. 1998).

In the present study, the AM uptake into subcutaneous (s.c.) AT should be examined. In addition, it should be investigated, whether the concentrations of the drugs stay stable in AT, and thus, if AT is suitable for PM detection of cannabinoids. For this purpose, the perimortem and PM distribution in AT of different anatomical regions and the time- and temperature-dependent concentration changes of the cannabinoids and their main metabolites should be assessed.

## Materials and methods

### Chemicals and reagents

Formic acid and HPLC grade water were purchased from VWR-International (Darmstadt, Germany), and ethanol p.a. and HPLC grade acetonitrile from Sigma-Aldrich (Steinheim, Germany). Methanolic solutions of THC (0.1 mg/mL), THC pharmaceutical grade for drug administration (Dronabinol, DAC 2008, 98.5% purity), JWH-210 (solid), and RCS-4 (solid) were purchased from THC Pharm (Frankfurt/Main, Germany), THC-d3 (98.9% purity, 0.07% THC-d0), 11-hydroxy-THC (HO-THC), HO-THC-d3 (99.6% purity, 0.17% HO-THC-d0), 11-nor-9-carboxy-THC (THC-COOH), and THC-COOH-d3 solution (99.5% purity, 0.02% THC-COOH-d0, 0.1 mg/mL each) were obtained from LGC/Promochem (Wesel, Germany), and methanolic solutions of JWH-210-d9 (1 mg/mL, ≥ 99% purity) and RCS-4-d9 (5 mg/mL, ≥ 99% purity), hydroxypentyl-RCS-4 (HO-RCS-4) solution (10 mg/mL in acetonitrile), hydroxypentyl-JWH-210 (HO-JWH-210, solid), JWH-210-pentanoic acid (JWH-210-COOH, solid), and RCS-4-pentanoic acid (RCS-4-COOH, solid) from Cayman Europe (Tallinn, Estonia). The German Federal Criminal Police Office (Wiesbaden, Germany) provided JWH-210 used for drug administration with a purity of 98.3%. RCS-4 (96% purity) was purchased as ‘research chemical’ from an internet provider.

### Calibrators for standard addition

As already described in previous studies (Schaefer et al. 2019, 2020), the preparation of standard stock solutions (1 mg/mL) was performed by dissolving 5 mg of each solid compound in 5 mL of ethanol. Concentrations of working standard solutions (0.001 mg/mL, 0.01 mg/mL, 0.1 mg/mL) were obtained by diluting the stock solutions or liquid reference standards with ethanol, respectively. The concentrations of the calibrators used for standard addition were 20, 40, and 60 ng/g. All solutions were stored at − 20 °C.

### Animals

As described previously (Schaefer et al. 2019, 2020), twelve domestic male pigs (Swabian Hall strain; body weight (BW) 40.5–49.8 kg) were used for the study. They had free access to tap water and daily standard chow. They were kept fasting a night before the experiment and had free access to water.

### Surgical procedures

Surgical procedures have previously been described (Schaefer et al. 2015, 2016, 2017a, b, 2018a, b, 2019, 2020). At first, the animals received an intramuscular injection of xylazine hydrochloride (2.5 mg/kg, Rompun; Bayer, Leverkusen, Germany), ketamine hydrochloride (30 mg/kg, Ursotamin; Serumwerk Bernburg, Bernburg, Germany), and atropine (1 mg, Braun, Melsungen, Germany). Analgosedation was performed with isoflurane (2–4%, Forene, AbbVie, Ludwigshafen, Germany), mechanical ventilation with a mixture of oxygen and air (1:2 vol/vol; FiO_2_ of 0.30; Respirator ABV-U; F. Stephan GmbH, Gackenbach, Germany) and volume cycling with a tidal volume of 10–12 mL/kg. A triple-lumen 7F (Certofix Trio, Braun, Melsungen, Germany) central venous catheter was inserted into the jugular vein for monitoring of mean central venous pressure. One catheter was set into the left ear vein to replace fluid (sodium chloride 0.9% [8 mL kg^−1^ h^−1^], Braun, Melsungen, Germany). To measure invasive blood pressure and blood gases, the left femoral artery was catheterized (Leadercath Expert 14G, Vygon, Aachen, Germany). Finally, a suprapubic catheter (Cystofix, Braun, Melsungen, Germany) was placed into the bladder for urine sample collection. The animals were then allowed to stabilize for 10–15 min.

### Study design

As already described elsewhere (Schaefer et al. 2018a, b, Schaefer et al. 2019, 2020), at first, a stock solution (7.5 mg/mL of JWH-210, RCS-4, and THC each) was prepared in ethanol. To obtain a 200 µg per kg BW dose, the appropriate volume of the solution (1080–1328 µL) was applied, respectively. The drugs were administered to 12 pigs within 12 min by nebulization using the inspiration-triggered mode (< 0.2 mL/min) of the M-neb flow^+^ ventilation ultrasonic nebulizer MN-300/7 (Nebutec, Elsenfeld, Germany) allowing for simulation of authentic user habits (Schaefer et al. 2018a, b).

Subcutaneous AT specimens were collected ventrally 15 and 30 min after administration and then hourly up to 8 h. As described previously (Schaefer et al. 2019), 8 h after administration (PMI 0), the animals were euthanized with T 61 (0.12 mL/kg BW, Intervet Deutschland GmbH, Unterschleißheim, Germany) and the abdominal cavity was opened. Specimens of perirenal AT were collected and the abdominal cavity was sutured. Additionally, dorsal AT was sampled from the back of the animals.

The animals were stored at room temperature (RT; *n* = 6) or 4 °C (*n* = 6) in a supine position and further specimens were collected after 24, 48, and 72 h (PMI 1–3) each as described above. All samples were stored at − 20 °C until analysis.

### Sample preparation

AT specimens were prepared according to Schaefer et al. (2014) applying slight modifications. An amount of 2 g AT was homogenized (1 amount tissue + 5 amounts acetonitrile). Subsequently, four 0.5-g portions were prepared with and without addition of 20 ng/g, 40 ng/g, or 60 ng/g of JWH-210, HO-JWH-210, JWH-210-COOH, RCS-4, HO-RCS-4, RCS-4-COOH, THC, 11-HO-THC, and THC-COOH to create a standard addition calibration curve. After centrifugation at 3500×*g* for 8 min, 1.5 mL of the supernatants were added to 20 µL of an ethanolic stable-isotope-labeled internal standard mixture solution (2 ng/20 µL of JWH-210-d9 and RCS-4-d9, 10 ng/20 µL of THC-d3) and evaporated under nitrogen at 60 °C. The dry residues were dissolved in 100 µL of mobile phase A/B (50:50, v/v). Mobile phase A was 0.1% aqueous formic acid and B consisted of 0.1% formic acid in acetonitrile. Twenty microliters were then injected onto the liquid chromatography tandem-mass spectrometry (LC–MS/MS) system.

### Standard addition method

For quantification of the drugs and their metabolites in AT specimens, the standard addition approach was chosen. Four portions were prepared of each specimen, one containing no calibrator solution and three containing different concentrations of calibrator solution. Subsequently, regression analysis was performed. Standard addition calibration equations were created as follows: *y* = *a x* + *b*. Depending on the slope (*a*) and the intercept (*b*) the calibration curve intersects the *x*-axis at the negative side. The point of intersection represents the unknown concentration.

### Apparatus

#### LC–MS/MS

LC–MS/MS conditions including instrumentation, chromatographic, and mass spectrometric conditions for the analysis of extracts have already been described elsewhere (Schaefer et al. 2015, 2017b, 2019, 2020). In brief, a Thermo Fisher (TF, Dreieich, Germany) HPLC was applied consisting of one Allegro pump and an HTC PAL autosampler. Applying a TF TSQ Quantum Ultra Accurate Mass triple stage mass spectrometer with an atmospheric pressure chemical ionization (APCI) interface running in the positive mode detection was achieved. The column was a Waters (Wexford, Ireland) Sunfire C_18_ column (150 × 2.1 mm, 3.5 µm). Gradient elution was carried out using mobile phase A and B. The runtime was about 10 min. Ionization was performed with the APCI source in positive mode and following settings: discharge current 5.0 µA; vaporizer temperature 400 °C; sheath gas 40 arbitrary units; auxiliary gas 15 arbitrary units; capillary temperature 270 °C. Detection and quantification of the compounds were performed in multiple-reaction monitoring mode with three transitions per precursor ion (Schaefer et al. 2018a, b). TF Xcalibur Version 2.0.7 SP 1 software was used.

#### Calculation of concentrations changes

In accordance to a recent study (Schaefer et al. 2020), the median concentration changes of the drugs (and their metabolites) determined in s.c., perirenal and dorsal AT specimens at PMI 1-3 in comparison with the median concentrations assessed right after death = PMI 0 were calculated using the following equation:

$$\Delta c \, \left( \% \right) = \left( {c\left( {{\text{PMI }}1 - 3} \right){-}c\left( {{\text{PMI }}0} \right)} \right)/c\left( {{\text{PMI }}0} \right) \times 100$$

A value higher than zero would indicate a concentration increase, a value lower than zero a concentration decrease.

### Statistical tests

For examination of the mean time-dependent concentration changes in the different AT specimens a non-parametric Friedman-test (*p* < 0.05) followed by the Dunn’s multiple comparison posthoc test was applied. A non-parametric Mann–Whitney *U*-test (*p* < 0.05) was carried out to compare concentrations determined after storage at RT with those determined after storage at 4 °C. Statistics were performed out using GraphPad Prism 5.00 (GraphPad Software, San Diego, CA, USA).

## Results

### Standard addition method

The drugs and their metabolites were quantified in AT specimens applying the standard addition method. Regression coefficients (*r*^2^) for JWH-210, RCS-4 and THC as well as HO-RCS-4 ranged between 0.95 and 0.99.

### AM disposition

JWH-210, RCS-4 and THC could be detected in every AM AT specimen of the 12 pigs. Mean maximum concentrations were reached in AM s.c. AT samples 0.5–2 h after administration and amounted to 21 ± 13 ng/g (JWH-210), 24 ± 13 ng/g (RCS-4), and 22 ± 20 ng/g (THC; see Table 1). In the following, concentrations stayed at the same level. After 8 h, concentrations were still at the plateau level (Table 1).

**Table 1:** Mean concentrations [± standard deviation (SD)] of JWH-210, RCS-4, HO-RCS-4 and THC in antemortem (AM) subcutaneous (s.c.) adipose tissue (AT) following a single pulmonary dose of 200 µg/kg body weight each to pigs (*n *= 12); concentrations are approximated and the mean of 12 values except for HO-RCS-4 (8 replicates); *n.d.* not detected

T (h)	Mean AM s.c. AT concentration ± SD (ng/g)			
	JWH-210	RCS-4	HO-RCS-4	THC
0.25	10 ± 8	13 ± 7	n.d.	14 ± 6
0.5	11 ± 6	19 ± 11	1 ± 1	22 ± 20
1	21 ± 13	22 ± 17	3 ± 2	18 ± 14
2	15 ± 10	24 ± 13	2 ± 1	22 ± 20
3	18 ± 9	23 ± 12	3 ± 2	17 ± 9
4	23 ± 17	21 ± 13	3 ± 2	20 ± 14
5	22 ± 20	19 ± 9	3 ± 2	20 ± 8
6	18 ± 12	19 ± 13	2 ± 1	18 ± 11
7	22 ± 24	17 ± 13	2 ± 2	20 ± 18
8	16 ± 5	20 ± 9	1 ± 1	20 ± 13

Regarding the metabolites, only very low concentrations of HO-RCS-4 with comparably high variabilities were detected in 8 pigs from 0.5 to 8 h (Table 1). No further metabolites were found in the analyzed AM s.c. AT specimens.

### PM concentrations and time- and temperature-dependent concentration changes

In general, high interindividual differences were observed in the analyzed PM specimens at PMI 0–3 under two different storage conditions. The median and mean concentrations (and their standard deviations; SD) of the parent compounds and the metabolite HO-RCS-4 are listed in Table 2. JWH-210, RCS-4 and THC could be detected in every PM AT specimen. At the time of death, mean concentrations of JWH-210 were highest in perirenal AT and lowest in dorsal AT (Table 2). Mean concentrations of RCS-4 were similar in s.c., perirenal and dorsal AT (Table 2). As for THC, lowest mean concentrations were determined in dorsal AT, whereby similar concentrations were found in s.c. and perirenal AT (Table 2).

**Table 2:** Median and mean [± standard deviation (SD)] concentrations of JWH-210, RCS-4, HO-RCS-4 and THC in subcutaneous (s.c.), perirenal and dorsal adipose tissue (AT) measured at postmortem interval (PMI) 0-3 of pigs stored at 4 °C or room temperature (RT); concentrations are approximated

	JWH-210 Median conc. [Mean conc. ± SD] in ng/g								RCS-4 Median conc.[Mean conc. ± SD] in ng/g							
	RT				4 °C				RT				4 °C			
	PMI 0	PMI 1	PMI 2	PMI 3	PMI 0	PMI 1	PMI 2	PMI 3	PMI 0	PMI 1	PMI 2	PMI 3	PMI 0	PMI 1	PMI 2	PMI 3
s.c. AT	14 [14 ± 4] (*n* = 6)	15 [16 ± 6] (*n* = 6)	20 [38 ± 34] (*n* = 12)	21 [17 ± 10] (*n* = 6)	16 [18 ± 6] (*n* = 6)	8 [11 ± 9] (*n* = 6)	18 [17 ± 12] (*n* = 6)	11 [12 ± 7] (*n* = 6)	22 [21 ± 12] (*n* = 6)	18 [16 ± 6] (*n* = 6)	26 [29 ± 21] (*n* = 6)	27 [32 ± 18] (*n* = 6)	18 [19 ± 6] (*n* = 6)	39 [39 ± 26] (*n* = 6)	22 [22 ± 8] (*n* = 6)	22 [27 ± 19] (*n* = 6)
Perirenal AT	26 [25 ± 9] (*n* = 6)	19 [26 ± 19] (*n* = 6)	17 [16 ± 8] (*n* = 6)	14 [27 ± 29] (*n* = 6)	18 [22 ± 17] (*n* = 6)	23 [20 ± 13] (*n* = 6)	21 [17 ± 6] (*n* = 6)	12 [17 ± 11] (*n* = 6)	16 [15 ± 4] (*n* = 6)	18 [20 ± 8] (*n* = 6)	22 [20 ± 4] (*n* = 6)	31 [33 ± 16] (*n* = 6)	19 [25 ± 25] (*n* = 6)	19 [28 ± 25] (*n* = 6)	19 [28 ± 18] (*n* = 6)	41 [41 ± 17] (*n* = 6)
Dorsal AT	10 [10 ± 5] (*n* = 6)	21 [24 ± 15] (*n* = 6)	18 [19 ± 3] (*n* = 6)	19 [20 ± 6] (*n* = 6)	10 [16 ± 19] (*n* = 6)	14 [16 ± 10] (*n* = 6)	13 [20 ± 17] (*n* = 6)	10 [18 ± 21] (*n* = 6)	14 [17 ± 9] (*n* = 6)	13 [12 ± 6] (*n* = 6)	20 [20 ± 7] (*n* = 6)	19 [28 ± 24] (*n* = 6)	27 [20 ± 13] (*n* = 6)	16 [22 ± 13] (*n* = 6)	22 [23 ± 11] (*n* = 6)	13 [14 ± 9] (*n* = 6)

	HO-RCS-4 Median conc. [Mean conc. ± SD] in ng/g								THC Median conc. [Mean conc. ± SD] in ng/g							
	RT				4 °C				RT				4 °C			
	PMI 0	PMI 1	PMI 2	PMI 3	PMI 0	PMI 1	PMI 2	PMI 3	PMI 0	PMI 1	PMI 2	PMI 3	PMI 0	PMI 1	PMI 2	PMI 3
s.c. AT	1 [1 ± 0] (*n* = 6)	5 [4 ± 2] (*n* = 6)	5 [5 ± 2] (*n* = 6)	2 [2 ± 1] (*n* = 6)	2 [2 ± 1] (*n* = 3)	4 [8 ± 10] (*n* = 3)	1 [3 ± 3] (*n* = 3)	2 [2] (*n* = 2)	18 [27 ± 25] (*n* = 6)	20 [21 ± 9] (*n* = 6)	30 [28 ± 10] (*n* = 6)	17 [18 ± 9] (*n* = 6)	15 [14 ± 8] (*n* = 6)	16 [18 ± 10] (*n* = 6)	13 [13 ± 5] (*n* = 6)	10 [13 ± 5] (*n* = 6)
Perirenal AT	1 [1 ± 1] (*n* = 5)	9 [13 ± 12] (*n* = 5)	15 [14 ± 10] (*n* = 5)	10 [44 ± 75] (*n* = 5)	4 [5 ± 3] (*n* = 5)	10 [10 ± 7] (*n* = 5)	7 [10 ± 11] (*n* = 5)	20 [17 ± 16] (*n* = 5)	15 [19 ± 15] (*n* = 6)	14 [19 ± 13] (*n* = 6)	13 [17 ± 14] (*n* = 6)	25 [35 ± 23] (*n* = 6)	16 [20 ± 13] (*n* = 6)	11 [17 ± 17] (*n* = 6)	28 [24 ± 16] (*n* = 6)	23 [22 ± 9] (*n* = 6)
Dorsal AT	2 [3 ± 2] (*n* = 3)	1 [1 ± 0.5] (*n* = 3)	3 [3 ± 3] (*n* = 3)	4 [3 ± 2] (*n* = 3)	2 [3 ± 2] (*n* = 4)	5 [4 ± 3] (*n* = 4)	3 [3 ± 2] (*n* = 4)	3 [3 ± 1] (*n* = 4)	8 [13 ± 12] (*n* = 6)	15 [15 ± 5] (*n* = 6)	19 [18 ± 7] (*n* = 6)	16 [19 ± 13] (*n* = 6)	8 [9 ± 9] (*n* = 6)	10 [12 ± 5] (*n* = 6)	12 [13 ± 6] (*n* = 6)	12 [13 ± 6] (*n* = 6)

**Fig. 2:**
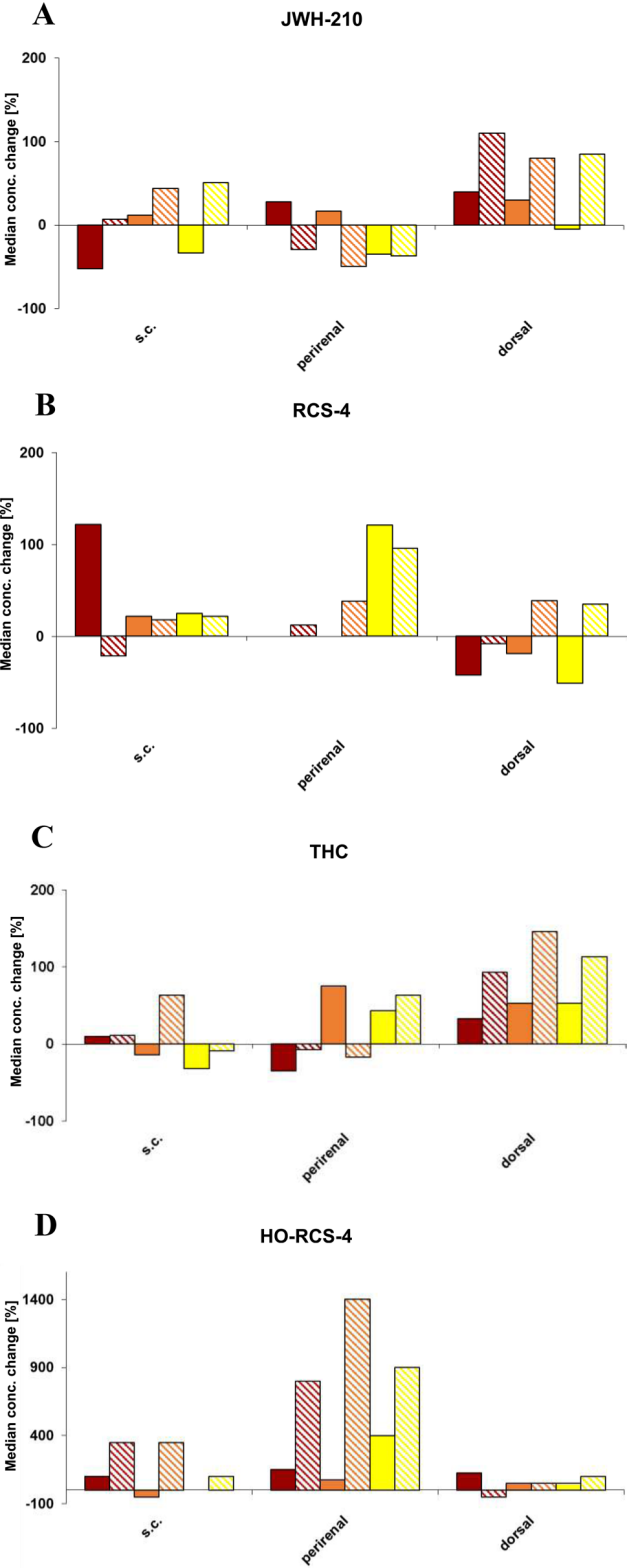
Time- and temperature-dependent postmortem concentration changes of **a** JWH-210, **b** RCS-4, **c** THC and **d** HO-RCS-4.  PMI 1, (4 °C);  PMI 1, (RT);  PMI 2, (4 °C);  PMI 2, (RT);  PMI 3, (4 °C);  PMI 3, (RT); in pig subcutaneous (s.c.), perirenal and dorsal adipose tissue (AT) following pulmonary administration of a 200 μg/kg body weight dose each. Concentrations are displayed as the median concentration change compared to concentrations calculated at PMI 0; *RT* room temperature

PM mean concentrations of parent compounds in s.c., perirenal, and dorsal AT did not change significantly (*p* > 0.05) over time under both storage conditions (Table 2). Median concentration changes are depicted in Fig. 2a–c. Regarding JWH-210, only minor differences were observed of concentrations at PMI 1–3 as compared to PMI 0 throughout the tested specimens. Concentrations increased only slightly in dorsal AT stored at RT (Fig. 2a, Table 2). Concerning RCS-4, slight continuous concentration increases were assessed in perirenal AT during the whole observation period (storage at RT and 4 °C each, see Fig. 2b, Table 2). However, the concentrations in dorsal AT decreased during storage at 4 °C (Fig. 2b, Table 2). With regard to THC, a slight increase of concentrations determined in dorsal AT of pigs stored at RT was observed (Fig. 2c, Table 2).

As for the detectability of metabolites in PM AT specimens, only HO-RCS-4 could be found. This metabolite was detected in s.c. AT specimens of every pig stored at RT and of 3 out of 6 pigs stored at 4 °C, respectively (see Table 2). In perirenal AT specimens, HO-RCS-4 was detected in 5 of 6 pigs stored at RT and 4 °C, respectively. In dorsal AT specimens, the metabolite was identified in 4 of 6 animals stored at 4 °C and in 3 of 6 animals stored at RT, respectively (see Table 2). No significant changes of mean PM concentrations could be observed except for concentrations determined in perirenal AT showing a significant (*p* < 0.05) time-dependent increase of HO-RCS-4 in specimens stored at RT (Table 2). Median concentration changes are depicted in Fig. 2d.

## Discussion

### Dosage

The total doses of JWH-210, RCS-4, and THC administered inhalatively amounted to 8.1–9.9 mg. These dosages were comparable with SC doses used in controlled animal studies or human self-experiments (Castaneto et al. 2015) as well as THC doses applied in human studies with inhalative consumption (Desrosiers et al. 2014; Hazekamp et al. 2006).

### Standard addition method

As already discussed elsewhere (Schaefer et al. 2020), the standard addition approach is more labor-intensive as compared to the conventional method validation, but matrix effects are leveled (Jickells and Negrusz 2008) because of matrix-matched calibration curves. Especially in PM toxicology this issue is of importance, as matrix effects can be challenging due to purification of specimens. In addition, in common validation procedures the use of blank matrix from different individuals is demanded for the assessment of several parameters. However, in case of PM specimens this might lead to unrepresentative results, since interindividual biological variances of the same matrix specimens have to be considered. For this purpose, national and international guidelines recommend the application of the standard addition method for quantification of drugs in (PM) tissue specimens (GTFCh 2018; Jickells and Negrusz 2008; Peters et al. 2007; Skopp 2010; SOFT/AAFS 2006).

Taking into account the high purity of the stable-isotope-labeled internal standards guaranteed by the vendor, no relevant interferences with the analytical results had to be expected. As described previously (Schaefer et al. 2020), the concentrations of the calibrators were determined according to a rough semiquantitative estimation of the amount found in initial analyses. The calibrator concentrations were accordingly adjusted. The calibration curves were regarded to be linear with *r*^2^ > 0.95.

### AM distribution

The results suggest a rapid uptake of the parent compounds into s.c. AT with THC most rapidly reaching the maximum concentrations (Table 1). The fact that RCS-4 concentrations in s.c. AT specimens peaked latest after 2 h might be explained by a lower lipophilicity (Table 3) and a much faster metabolization rate as compared to the other two cannabinoids (Schaefer et al. 2018a, b).

**Table 3:** Data on basicity [pk_a_, (ChemIDplus 2020)], lipophilicity (logP, according to Schaefer et al. (2017b)), ratio of maximum concentration in adipose tissue (*C*_at_) and corresponding concentration in serum (*C*_se_) according to Schaefer et al. (2019) and adipose tissue storage index (ASI) calculated as a quotient of *C*_at_ and relative administered dose of JWH-210, RCS-4, and THC; *n.a.* not available

Cannabinoid	pk_a_	logP	*C*_at_/*C*_se_	ASI
JWH-210	n.a.	7.5	1.7	0.10
RCS-4	n.a.	5.6	5.7	0.12
THC	10.6	6.7	1.0	0.11

The data of the current study further indicate a persistence of the parent drugs in s.c. AT for the duration of the experiment, because quite similar concentrations were observed over the entire observation period until the end of the experiment (Table 1). These findings are in line with our previous study providing data on the TK properties of the parent drugs in pig serum (Schaefer et al. 2018a, b). The fact that the drug concentrations in serum rapidly reached their maximum a few minutes after the beginning of the inhalative administration followed by a steep decrease (see also the ratio of the maximum concentration in AT to the corresponding concentration in serum; Table 3) was explained by a very immediate distribution into deep compartments (Schaefer et al. 2018a, b). This explanatory approach was further reinforced by the high volumes of distribution calculated based on the data of that TK study (Schaefer et al. 2018a, b).

In line with our findings, a rapid uptake of THC into AT has already been discussed, e.g., by Rawitch et al. (1979). These authors assessed maximum concentrations in mouse gonadal AT a few minutes after intraperitoneal injection. The concentrations also dropped to a plateau level that was maintained for at least 6 h.

Regarding SCs, no data on their time-course of uptake into AT are available. However, in some TK studies on blood concentration–time profiles a distribution into AT and a sequestration in this tissue was at least discussed hypothetically. One study was performed with the two so-called ‘new generation’ SC CUMYL-PICA and 5F-CUMYL-PICA using rats (Kevin et al. 2017). The authors showed a similar time-course of distribution in plasma than we found in our TK study (Schaefer et al. 2018a, b). To explain the slow clearance from plasma, they discussed a possible persistence in and redistribution from AT. As a consequence, SC could be supposed to passively and slowly diffuse into blood again during abstinence or food deprivation (Kevin et al. 2017). This hypothesis was also supported by Franz et al. (2020) assessing an extraordinary long detection window of a SC metabolite in human urine. The aforementioned phenomena have already been described for THC (Gunasekaran et al. 2009; Johansson et al. 1989), leading to the conclusion that a storage in AT might alter the TK of a drug being an important issue with regard to abstinence control programs. As a consequence, TD, that is to say, the psychotropic drug effects might be influenced.

When determining concentrations in AT as a function of time, possible mechanisms of uptake into adipocytes have to be discussed. The most important issue might be the lipophilicity of a drug, expressed as a high octanol/water coefficient (logP). Thiopental with a logP value of 2.8 is said to be a prototype of a ‘fat-seeker’ accumulating with high affinity in AT (Mühlebach et al. 1985). According to an in vitro study by Betschart et al. (1988), evidence is provided that those drugs with a logP value below 2 are not supposed to be stored in AT. However, some highly lipophilic drugs such as methadone, imipramine, amitriptyline and chlorpromazine are not significantly taken up by adipocytes in vivo (Betschart et al. 1988; Mühlebach et al. 1985), implicating that distribution into AT is not simply a matter of lipid solubility. In fact, Mühlebach et al. (1985) demonstrated that the rates of invasion into AT considerably decrease with increasing lipophilicity. Particularly, the chemical structure of basic lipophilic drugs seems to play an important role concerning the extent of AT storage (Moor et al. 1992). In this context, high affinity binding to rapidly perfused organs due to lysosomal trapping (also called lysosomotropism) might be the underlying mechanism (Macintyre and Cutler 1988). Xie et al. (1991) investigated the AT uptake and storage of benzodiazepines with different basic properties (pk_a_) and drew the conclusion that drugs with pk_a_ < 7 are likely to be stored in AT and those with pk_a_ > 7 show no considerable persistence.

For the drugs analyzed in the present study, no data are available concerning pk_a_ values of JWH-210 and RCS-4. Those substances contain a tertiary amine located in an indole core resulting in only weak basic properties. THC has a pk_a_ of about 10 [Table 3; (ChemIDplus 2020)] suggesting only low storage affinity. However, as a substance with a phenol moiety, it has weak acidic properties because of the mesomerism-stabilized anion of the phenolate. As for the lipophilic properties of the cannabinoids, logP values (see Table 3) are higher than the logP values of the studied benzodiazepines (Xie et al. 1991) and even higher than the logP of the ‘fat-seeker’ prototype thiopental (Mühlebach et al. 1985). However, the AT storage indices are consistently < 1 (Table 3) implicating a low distribution into AT as compared to other tissues. Unfortunately, specimens of the other tissues and organs were only sampled at the end of the experiment. Thus, the AM kinetics in AT could not be compared with concentration–time profiles in other tissues. However, the time-course of concentrations in s.c. AT indicate a stable retention in this tissue.

The analyses of metabolites in AM AT specimens indicated that only HO-RCS-4 was found. Although being more lipophilic than HO-RCS-4, the main metabolites of the other two cannabinoids might not be distributed into AT, because they are not eliminated in their free form, but as highly hydrophilic glucuronides (Schaefer et al. 2018a, b). Since no enzymatic cleavage was performed in this study, only the free metabolites, but not the glucuronides could be detected.

### PM concentrations and time- and temperature-dependent concentration changes

At the time of death (8 h after administration), the concentrations of the parent drugs in s.c. AT had not changed considerably as compared to the maximum AM AT concentrations. The concentrations in perirenal AT were resembled to those measured in s.c. AT except for JWH-210 exhibiting the highest concentrations in this region (Table 2). This might be explained by the fact that JWH-210 is supposed to accumulate in kidneys (Schaefer et al. 2017b, 2019). Thus, a diffusion into the surrounding perirenal AT seems possible. In accordance with previously published data (Schaefer et al. 2017b), lowest drug concentrations were observed in dorsal AT. This distribution might be explained by the thicker texture and a minor supply with blood vessels (Schaefer et al. 2017b).

Comparing the concentrations in AT at the time of death with those in the remaining tissues and organs (Schaefer et al. 2019), higher mean concentrations of JWH-210 were found in lung (33 ± 19 ng/g) and kidney (25 ± 41 ng/g) (Schaefer et al. 2019). As a consequence, besides AT, an additional sequestration of JWH-210 seems to occur into lung and kidney. Concerning RCS-4, the concentrations in the other tissues were lower as compared to those in AT except for lung. In this organ, even slightly higher concentrations (20 ± 19 ng/g) were determined (Schaefer et al. 2019), indicating a comparable persistence of RCS-4 in these tissues. As for THC, concentrations were comparably high in liver (26 ± 20 ng/g) and bile fluid (25 ± 47 ng/g) and much higher in duodenum content (84 ± 66 ng/g) (Schaefer et al. 2019). However, this discrepancy can be explained by the fact that the sample preparation in the former study included an enzymatic cleavage. Thus, the total amount of THC and THC-glucuronide was detected, leading to higher concentrations in duodenum content due to enterohepatic circulation.

In addition, PM concentrations of the drugs exhibited relevant PM changes in these organs over an observation period of 72 h (Schaefer et al. 2020). On the contrary, only minor PM concentration changes (*p* > 0.05) of the parent drugs were encountered throughout the tested AT specimens, irrespective of storage time and temperature (Fig. 2a–c, Table 2). The minor concentration changes (about 100%) found might be attributable to interindividual and analytical variations. At least, the slight continuous concentration increase of RCS-4 in perirenal AT might be a result of PMR from the kidneys. The concentration increase of RCS-4 in s.c. AT at PMI 1 (4 °C) might be explained by one pig exhibiting great intra-individual variability (see Fig. 2b, Table 2). These findings indicate a PM stability of the drugs in AT with concentrations not considerably being altered by so-called PMR phenomena. A resistance of AT towards PMR has already been discussed elsewhere (Levisky et al. 2001). As far as THC is concerned, Brunet et al. (2010), studying the time-dependent PMR of THC in pigs following intravenous administration of a 200 µg/kg BW dose, provided similar data.

Regarding SC, scientific data on their distribution are very sparse. However, many fatal case reports have been published (Castaneto et al. 2015; Kraemer et al. 2019; Meyer 2016). As far as analytical data on AT distribution were provided, comparable or even much higher SC concentrations were reported (Castaneto et al. 2015; Kraemer et al. 2019). The interpretation of the differences is difficult yet, because time and dose of intake or the PMI as well as consumption habits were unknown. In those studies with comparable SC AT concentrations, a similar distribution pattern in the remaining organs and tissues was observed as in our study (Schaefer et al. 2019).

With respect to HO-RCS-4, concentrations in AT after 8 h were lower as compared to those detected in the remaining tissues (Schaefer et al. 2019), suggesting a minor AT storage potential.

The concentrations of the metabolite in s.c. and dorsal AT seemed to be stable at least over 72 h (Fig. 2d). However, in perirenal AT a significant time-dependent increase (*p* < 0.05) was observed during storage at RT (Fig. 2d). This phenomenon might again be explained by the vicinity of perirenal AT to the kidneys.

## Conclusion

Antemortem distribution patterns and PM time- and temperature-dependent concentration changes of the two SC JWH-210 and RCS-4 as well as THC following pulmonary administration were investigated in pigs. The results suggest a rapid distribution of the cannabinoids into s.c. AT and a sequestration in this tissue within the observation period of 72 h allowing for redistribution. This issue has to be considered in the context of abstinence control analyses, especially with regard to users with chronic consumption habits. In addition, the findings indicate that AT might be resistant to PMR of parent compounds and, therefore, viable as alternative matrix in PM toxicology. As for metabolites, the less lipophilic HO-RCS-4 was the only metabolite detected in AT specimens, probably as the other analyzed metabolites are only distributed and eliminated as glucuronides. Last but not least, significant PM increases of metabolite concentrations might be considered in perirenal AT due to immediate vicinity to the kidneys.
